# Improving antibiotic prescribing in LMICs: Insights from an outpatient clinic in Pakistan

**DOI:** 10.1016/j.nmni.2025.101680

**Published:** 2025-12-02

**Authors:** Tamim Khawaja, Mikael Kajova, Anu Kantele

**Affiliations:** a Department of Infectious Diseases, Inflammation Centre, University of Helsinki and Helsinki University Hospital, P.O. Box 340, 00029 HUS, Finland; b Human Microbiome Research Program, University of Helsinki, P.O. Box 63, 00014, Finland; c Meilahti Vaccine Research Centre, University of Helsinki and Helsinki University Hospital, P.O. Box 340, 00029 HUS, Finland; d FIMAR, Multidisciplinary Centre of Excellence in Antimicrobial Resistance Research, University of Helsinki, P.O. Box 3, 00014, Finland

**Keywords:** Antimicrobial resistance (AMR), Antibiotic stewardship, Outpatient prescribing, Low- and middle-income countries (LMICs), Pakistan, Guideline adherence, Resource-limited settings

## Abstract

**Background:**

Most human antibiotic use occurs in outpatient care and is a key driver of antimicrobial resistance (AMR). Although sales statistics suggest that high-income countries consume more antibiotics overall, the steepest growth is seen in low- and middle-income countries. Sales data – incomplete and non-transparent – reveal little about prescriptions to individual patients. Detailed, clinic-level data are essential for identifying targets for stewardship.

We investigated antibiotic use and prescription appropriateness in an outpatient clinic in Pakistan – a country with some of the world's highest reported AMR prevalences.

**Methods:**

Patients attending an outpatient clinic near Lahore were interviewed immediately after their clinic visit and again one month later. Clinical data were collected through patient interviews, and antibiotic prescriptions were extracted from patient records.

**Results:**

Of the 983 participants, 398 (40.5 %) were prescribed antibiotics at the primary visits. Metronidazole was the single most common agent, followed by first-generation cephalosporins, fluoroquinolones, and amoxicillin–clavulanate; phenoxymethylpenicillin and amoxicillin were only minimally prescribed. One-third of prescriptions (33.2 %) were combination regimens, 90.2 % of which included metronidazole. Guideline-concordant first- or second-line choices accounted for only 23.4 % of prescriptions.

Of all patients, 493 (50.2 %) attended a one-month control visit. Of these, 233 (47.3 %) also saw a practitioner during the visit; 73 of them (31.3 %) were prescribed antibiotics.

**Conclusions:**

Antibiotic prescribing was excessive and discordant with recommended first-line therapies, favouring broad-spectrum agents and combination treatments – practices likely influenced by the negligible time available per patient. Our findings highlight the need to adapt antimicrobial stewardship interventions to limited-resource contexts.

## 1 Background

Antimicrobial resistance (AMR) is a global health crisis, with most severe consequences in low- and middle-income countries (LMICs), where it is associated with millions of deaths annually [1]. The primary driver of this crisis is the overuse and misuse of antibiotics [2] exacerbated by weak policies regulating their use in human healthcare and agriculture [3,4]. One of the main targets of global AMR containment are antibiotic overprescription and inappropriate use in LMICs. However, data on antibiotic usage in LMICs remain limited.

South Asia is among the regions hardest hit by the AMR crisis [1,[3], [4], [5]], driven by factors such as high population density, poverty, poor hygiene, and inadequate healthcare systems [4,5]. Antibiotics are cheap and readily available even over-the-counter [6] and, moreover, physicians receive incentives from the industry when prescribing them [4,7].

We investigated AMR in Pakistan with two separate approaches. Our recent report addressed *E. coli* colonisation diversity and resistance genes [8]. Here, we examine real-life antibiotic use in the same settings, showing that prescribers frequently select antibiotics and antibiotic combinations with unnecessarily broad or suboptimal coverage. We suggest concentrating stewardship efforts more on antibiotic selection rather than prescribing frequency.

## 2 Methods

### 2.1 Study design

We explored antibiotic prescribing practices at the outpatient clinic of the Punjab Social Security Health Management Company Hospital Raiwind (henceforth Manga Mandi Hospital). Data on antibiotic prescriptions and symptoms were collected through electronic patient records and structured interviews conducted on-site by the researchers. Participants were invited to a follow-up visit one month after the initial visit. Rectal swabs/stool samples were collected at enrolment and follow-up visits; *E. coli* diversity and AMR findings have been published previously [8].

## 3 Manga Mandi outpatient clinic

Located in an industrial area within greater Lahore, the hospital serves 83,000 workers and 113,000 dependents. Funded by employers, it offers free consultations and medications with visits possible during working hours. The walk-in outpatient clinic is staffed by approximately 10 doctors, mainly providing primary care. In the year preceding our study, the clinic recorded 399,000 visits. With estimated 300 working days and five physician/dentist hours/day, this amounts to 26.6 patients/hour/practitioner or 2.3 min/consultation. Most visits concerned minor ailments.

### 3.1 Recruitment

Patients were recruited between February–June 2016, immediately after their appointments and without selection. Their doctors directed them to the study site next door. Due to the clinic's fast pace, refusals could not be tracked. After consent, participants were interviewed and invited to a follow-up visit after 30 days [8].

### 3.2 Data collection

The structured interviews (Table 1) were conducted in participants’ native language.

**Table 1:** Demographic and clinical characteristics of study population.

	Primary visit only	Both visits attended	All
**Number of participants**, *n* (%)	490 (49.8)	493 (50.2)	983 (100)
**Sex**, *n* (%)			
Male	253 (51.6)	235 (47.7)	488 (49.6)
Female	237 (48.4)	258 (52.3)	495 (50.4)
**Age group**, *n* (%)^a^			
<12 months	3 (0.6)	1 (0.2)	4 (0.4)
12–23 months	4 (0.8)	1 (0.2)	5 (0.5)
2–13 years	38 (7.8)	39 (7.9)	77 (7.8)
14–64 years	411 (83.9)	426 (86.4)	837 (85.1)
≥65 years	34 (6.9)	26 (5.3)	60 (6.1)
**Socioeconomic Class**, *n* (%)			
Working class	479 (97.8)	484 (98.2)	963 (98.0)
Lower middle class	8 (1.6)	8 (1.6)	16 (1.6)
Middle class	3 (0.6)	1 (0.2)	4 (0.4)
**Hospitalised past 12 mo**, *n* (%)	29 (5.9)	23 (4.7)	52 (5.3)
**Updated CCI points**^b^, *n* (%)			
0	460 (93.9)	444 (90.1)	904 (92.0)
1	26 (5.3)	41 (8.3)	67 (6.8)
2	4 (0.8)	8 (1.6)	12 (1.2)
**Diabetes**, *n* (%)	31 (6.3)	50 (10.1)	81 (8.2)
**Regular medication**, *n* (%)	103 (21.0)	145 (29.4)	248 (25.2)
**Reason(s) for visit**, *n* (%)^c^^d^			
ENT problem (common cold, wax)	155 (31.6)	163 (33.1)	318 (32.3)
General weakness or pains	121 (24.7)	108 (21.9)	229 (23.3)
Gastroenterological (constipation, diarrhoea)	114 (23.3)	90 (18.3)	204 (20.8)
Musculoskeletal (back pain, limb pain)	41 (8.4)	51 (10.3)	92 (9.4)
Dermatological	40 (8.2)	33 (6.7)	73 (7.4)
Diabetes or hypertension control visit	24 (4.9)	43 (8.7)	67 (6.8)
Dental	36 (7.3)	30 (6.1)	66 (6.7)
Pulmonological (cough, asthma)	30 (6.1)	33 (6.7)	63 (6.4)
Fever	27 (5.5)	16 (3.2)	43 (4.4)
Other internal medicine reason	13 (2.7)	12 (2.4)	25 (2.5)
Chronic viral hepatitis	9 (1.8)	13 (2.6)	22 (2.2)
Gynecological	8 (1.6)	8 (1.6)	16 (1.6)
Ophthalmological	6 (1.2)	10 (2.0)	16 (1.6)
Gastrosurgical (hernia, anal fissure)	5 (1.0)	9 (1.8)	14 (1.4)
Neurological	9 (1.8)	5 (1.0)	14 (1.4)
Other infection (cellulitis, tuberculosis)	4 (0.8)	6 (1.2)	10 (1.0)
UTI or other urinary issue	6 (1.2)	6 (1.2)	12 (1.2)

a Especially older volunteers did not know their exact age and provided their best estimate.

b Updated Charlson comorbidity index (Quan H et al. Am J Epidemiol. 2011; 173(6):676–82.).

c Typical issues are shown in parentheses.

d Patients often consulted more than one physician for different reasons during the same clinic visit, therefore, the number of reasons for visit exceeds the total number of patients.

The electronic patient records included all prescriptions but no clinical data. For the six months preceding the primary visit, we recorded the number of systemic antibacterial (henceforth antibiotics) courses and the date and type of the most recent one. Antibiotics prescribed at the primary visit and between the visits were recorded in detail. At the follow-up visit, we recorded only whether antibiotics were prescribed. Antifungal and antiviral prescriptions were excluded. Simultaneously prescribed antibiotics were treated as a single course.

A Finnish infectious disease specialist (TK) evaluated indications for antibiotic use based on the reported symptoms, categorizing them as likely, possible, unlikely, or highly unlikely indications for antibiotics.

Antibiotic choice was further assessed for appropriateness: 1) first- or second-line choice, 2) justifiable in special circumstances, 3) inadequate pathogen coverage, 4) excessively broad-spectrum even in special circumstances, and 5) inconclusive (no clear rationale). This classification addressed only the choice of antibiotic – not whether it was clinically warranted.

### 3.3 Data analysis

Data were analysed using SPSS 28.0 software (IBM Corp., Armonk, NY, USA) and R version 4.3.2 (The R foundation, Vienna, Austria).

### 3.4 Data availability

Pseudonymised data are not openly available due to GDPR. They can be requested from the corresponding author, subject to institutional approval.

## 4 Results

### 4.1 Study population

Of the 993 patients recruited, 10 were excluded (missing antimicrobial information: 2; caregiver provided his own information: 1; not providing study materials: 7), leaving in the final study population 983 patients. Of these, 493 (50.2 %) attended the scheduled follow-up 30 days later (median 30 days, IQR 28–32) with antibiotic data available also for the follow-up period (Table 1).

### 4.2 Background characteristics

Most participants were industrial workers or their dependents, 98 % from the lowest socioeconomic tier. Most were aged 14–64, with few young children or elderly. The most common complaints were upper respiratory tract symptoms, general weakness, and gastrointestinal symptoms (Table 1).

### 4.3 Antibiotic prescriptions in the 6 months prior to the primary visit

In the six months before the visit, 571/983 (58.1 %) had been prescribed antibiotics; 236/571 (41.3 %) had received ≥3 courses. At enrolment, 199 (20.2 %) patients were either on antibiotics or had completed a course within the past two weeks (Table 2).

**Table 2:** Systemic antibiotic courses^a^ prescribed in the Manga Mandi outpatient clinic: within 6 months prior to the primary visit, at the primary visit, between visits, and at the control visit^b^.

	Primary visit only	Both visits attended	All
**Number of participants**, *n* (%)	490 (49.8)	493 (50.2)	983 (100)
**Courses in prior 6 months**, *n* (%)			
0	213 (43.5)	199 (40.4)	412 (41.9)
1–2	165 (33.7)	170 (34.5)	335 (34.1)
3–5	85 (17.3)	91 (18.5)	176 (17.9)
6–9	20 (4.1)	25 (5.1)	45 (4.6)
≥10	7 (1.4)	8 (1.6)	15 (1.5)
**Latest course ended ≤14 days before enrolment**, *n* (%)	90 (18.4)	109 (22.1)	199 (20.2)
**Prescribed antibiotics during primary visit**, *n* (%)	195 (39.8)	203 (41.2)	398 (40.5)
**Prescribed antibiotics between visits**, *n* (%)	NA	126 (25.6)	NA
**Number of courses between visits, patients***n* (%)			
0	NA	367 (74.4)	NA
1	NA	86 (17.4)	NA
2	NA	28 (5.7)	NA
3 or more	NA	12 (2.4)	NA
**Median time between visits**, days (IQR)	NA	30 (28–32)	NA
**Visited a physician the day of control visit**, *n* (%)	NA	233 (47.3)	NA
**Prescribed antibiotics the day of control visit**, *n* (%)	NA	73 (14.8)	NA

a Any antibiotics prescribed at the same time were considered one course, regardless of whether one or multiple agents were included.

b All figures are patients n (%). Denominators: 490 (primary visit only), 493 (attended follow-up), 983 (all patients).

### 4.4 Antibiotic prescriptions at primary visit: indications and appropriateness

**Fig. 1:**
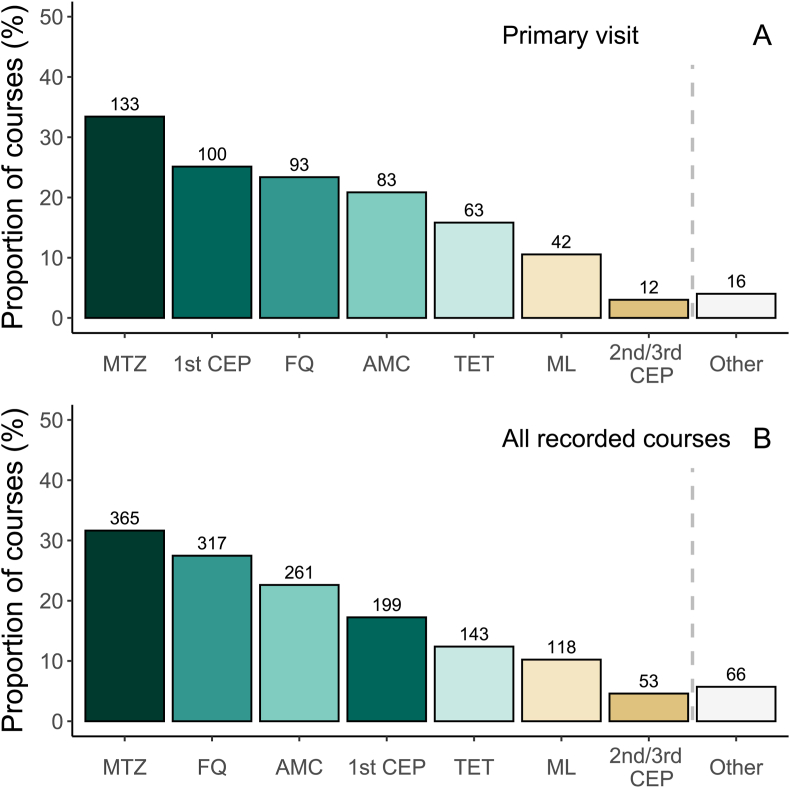
Most prescribed antibiotics, shown as the proportion of antibiotic courses containing each drug. **Panel A** presents seven most prescribed antibacterial agents in the 398 antibiotic courses prescribed at the primary visit to 983 participants. The bars depict the proportion of antibiotic courses containing each drug. The number above bars is the number of prescriptions containing the drug. **Panel B** includes all recorded antibiotic courses: the most recent course within six months prior to enrolment (571 courses among 983 patients), the 398 courses prescribed at the primary visit, and 185 courses prescribed between visits to 126 of the 493 patients who attended the one-month follow-up. In total, 1154 courses are shown. MTZ= metronidazole, 1st CEP= first generation oral cephalosporin, FQ= fluoroquinolone, AMC= amoxicillin–clavulanate, TET= tetracycline, ML= macrolide, 2nd/3rd CEP= second or third generation oral cephalosporin.

Antibiotics were prescribed to 398/983 patients (40.5 %), most commonly metronidazole (33 %), first-generation cephalosporins (26 %), fluoroquinolones (23 %), and amoxicillin–clavulanate (21 %); one third of prescriptions were combination therapies (Fig. 1, Table 3). Antibiotics were prescribed to 46.3 % of patients with a likely indication, 57.2 % with a possible, and 41.7 % with an unlikely and to 22.2 % with highly unlikely indication. Only 23 % of prescriptions were first- or second-line choices (Table 3).

**Table 3:** Antibiotic prescriptions on primary visit.

	Patients prescribed antibiotics, *n* = 398	Not prescribed antibiotics, *n* = 585
**Number of antibiotic courses prescribed**, *n*	398	
Number of combination courses, *n* (%)	132 (33.2)	
Total number of antibiotic agents prescribed, *n*	542	
**Need for antibiotics as judged by an investigator**, *n* (%)^a^		
Likely indication for antibiotics	6 (46.3)	7 (53.8)
Possible indication for antibiotics	174 (57.2)	130 (42.8)
Unlikely indication for antibiotics	150 (41.7)	210 (58.3)
Highly unlikely indication for antibiotics	68 (22.2)	238 (77.8)
**Prescriptions for patients with specific complaints**, *n* (%)^a^^b^		
Diarrhoea	15 (62.5)	9 (37.5)
Sore throat	58 (76.3)	18 (23.7)
Common cold or runny nose	55 (68.7)	25 (31.3)
Cough	18 (64.3)	10 (35.7)
Dental problem	41 (74.5)	14 (25.5)
**Guideline concordance of regimen**, *n* (%)^c^		
First- or second-line^d^	93 (23.4)	
Not in general, justified in exceptional circumstances^e^	100 (25.1)	
Probably not effective — wrong spectrum	76 (19.1)	
Too broad spectrum even in exceptional circumstances	76 (19.1)	
Cannot be evaluated due to the nature of complaints^f^	53 (13.3)	

a Percentages are within each indication category.

b Patients that had several complaints potentially requiring antibiotics, are not included here, however patients with cough and/or sore throat were included in the common cold/runny nose category if they had that complaint as well.

c Irrespective of whether antibiotics are normally indicated or not, for example amoxicillin–clavulanate would be considered 1st or second line choice for a patient who complains having a flu, since "flu" could be sinusitis. Percentages in this section are of those who were prescribed antibiotics.

d E.g. amoxicillin or 1st generation cephalosporin for a sore throat.

e E.g. a fluoroquinolone for upper respiratory symptoms.

f E.g. a macrolide for a patient with back pain.

To further assess prescribing patterns, we focused on five common complaints, selecting patients who presented with these symptoms alone, without coexisting conditions affecting antibiotic decisions. Prescription rates are summarized in Table 3, and drug choices in Table 4. Sore throat, common cold, and dental visit prescribing are further described in the discussion section.

**Table 4:** Number of antibiotic courses (%) prescribed for selected complaints on primary visit^a^.^b^.

Antibiotic	Runny nose/common cold	Sore throat	Cough	Dental issue	Diarrhoea
No antibiotics	25 (31.3)	18 (23.7)	10 (35.7)	14 (25.5)	9 (37.5)
1st CEP	15 (18.8)	24 (31.6)	3 (10.7)	0 (0.0)	1 (4.2)
ML	12 (15.0)	5 (6.6)	2 (7.1)	0 (0.0)	1 (4.2)
AMC	9 (11.3)	4 (5.3)	3 (10.7)	1 (1.8)	3 (12.5)
MTZ + AMC	3 (3.8)	10 (13.2)	1 (3.6)	11 (20.0)	0 (0.0)
MTZ + 1st CEP	1 (1.3)	1 (1.3)	1 (3.6)	15 (27.3)	0 (0.0)
MTZ + TET	2 (2.5)	2 (2.6)	0 (0.0)	10 (18.2)	0 (0.0)
FQ	7 (8.8)	7 (9.2)	4 (14.3)	0 (0.0)	0 (0.0)
MTZ + FQ	2 (2.5)	0 (0.0)	0 (0.0)	0 (0.0)	4 (16.7)
MTZ	0 (0.0)	1 (1.3)	0 (0.0)	1 (1.8)	3 (12.5)
FQ + MTZ + TET	0 (0.0)	0 (0.0)	0 (0.0)	2 (3.6)	0 (0.0)
TET	1 (1.3)	0 (0.0)	1 (3.6)	1 (1.8)	0 (0.0)
2nd/3rd CEP	0 (0.0)	1 (1.3)	1 (3.6)	0 (0.0)	1 (4.2)
MTZ + 2nd/3rd CEP	0 (0.0)	0 (0.0)	0 (0.0)	0 (0.0)	1 (4.2)
PEN	0 (0.0)	0 (0.0)	0 (0.0)	0 (0.0)	1 (4.2)
MTZ + AMC + TET	0 (0.0)	1 (1.3)	0 (0.0)	0 (0.0)	0 (0.0)
AMC + 1st CEP	0 (0.0)	1 (1.3)	0 (0.0)	0 (0.0)	0 (0.0)
FQ + ML	1 (1.3)	0 (0.0)	1 (3.6)	0 (0.0)	0 (0.0)
ML + 3rd CEP i.v.	1 (1.3)	0 (0.0)	1 (3.6)	0 (0.0)	0 (0.0)
MTZ + 1st CEP + 2nd/3rd CEP	0 (0.0)	1 (1.3)	0 (0.0)	0 (0.0)	0 (0.0)
3rd CEP i.v.	1 (1.3)	0 (0.0)	0 (0.0)	0 (0.0)	0 (0.0)
Total	80	76	28	55	24

Abbreviations of antimicrobials: 1st CEP = first-generation oral (p.o.) cephalosporins; ML = macrolides; AMC = amoxicillin–clavulanate; MTZ = metronidazole; TET = tetracyclines; FQ = fluoroquinolones; 2nd/3rd CEP = second- or third-generation oral (p.o.) cephalosporins; PEN = parenteral benzylpenicillin; 3rd CEP i.v. = third-generation parenteral cephalosporins (i.v. or i.m.).

a Patients included here had no other complaints affecting antibiotic choice except the one listed, the exception is "Runny nose/common cold" category into which those patients who also complained of cough and/or sore throat were included.

### 4.5 Antibiotic agents prescribed

For most commonly prescribed antibiotics, see Fig. 1: upper panel with prescriptions from the primary visit, lower panel with all prescriptions (most recent before enrolment, those at primary visit and during follow-up).

Metronidazole was the most frequently prescribed agent, included in over 30 % of all courses (Supplementary Tables 1 and 2). Other common antibiotics were first-generation cephalosporins, fluoroquinolones, amoxicillin–clavulanate, tetracyclines, and macrolides. Penicillins without a beta-lactamase inhibitor, clindamycin, nitrofurantoin, trimethoprim, and trimethoprim-sulphonamide combinations were absent or nearly so (Supplementary Table 1).

**Fig. 2:**
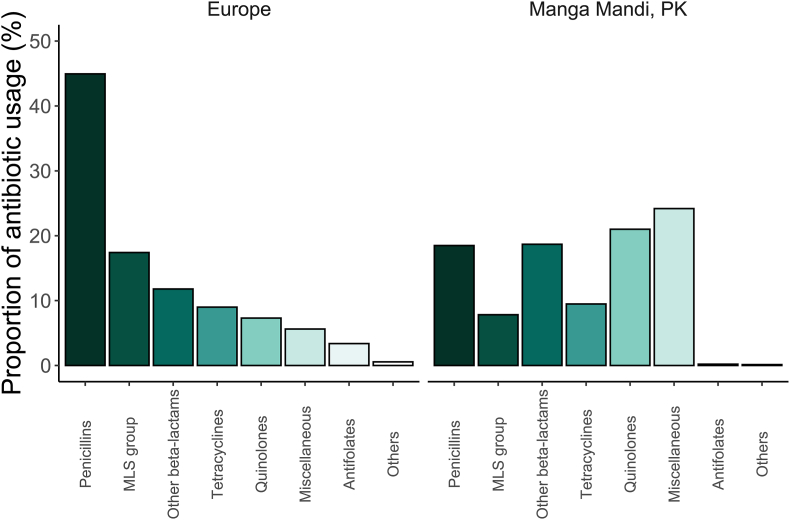
Antibiotic groups prescribed in Manga Mandi clinic compared with ECDC community prescribing data. Proportions of systemic antibiotics prescribed in Manga Mandi outpatient clinic (ATC level 3^a^) compared with 2022 ECDC community prescribing data [19]. Percentages for Manga Mandi are based on prescription counts/agent, whereas ECDC percentages are based on defined daily doses (DDD) per 1000 inhabitants per year. Manga Mandi data include all antibiotic prescriptions recorded in full: the most recent in the 6 months preceding enrolment, as well as prescriptions at the primary visit and during follow-up. Footnote to figure: ^a^The MLS group (J01F) comprises macrolides, lincosamides, and streptogramins. The miscellaneous group (J01X) includes diverse agents such as metronidazole, nitrofurantoin, fosfomycin, linezolid, and vancomycin. In Manga Mandi, only macrolides (from J01F) and metronidazole (from J01X) were prescribed. The “others” category includes J01B (amphenicols, e.g. chloramphenicol), J01G (aminoglycosides), and J01R (combination preparations of different antibacterial classes); none were prescribed in Manga Mandi.

Fig. 1 presents the proportion of courses including each antibiotic, while Fig. 2 uses total antibiotic counts as the denominator to allow comparison with ECDC community data. For numerical data, see Supplementary Table 1.

### 4.6 Prescriptions at follow-up visit

The follow-up visit was attended by 493 patients (median visit interval 30 days, IQR 28–32); 233 (47.3 %) opted to see a physician during the visit, and 73 (31.3 %) were prescribed antibiotics (Table 2).

## 5 Discussion

Our study highlights excessive and inappropriate prescribing of antibiotics in a small outpatient clinic in Pakistan.

### 5.1 Prescriptions in the 6 months before enrolment

Of all patients, 58.1 % (571/983) had been prescribed antibiotics during the 6 months prior to enrolment. This exceeds the 30.3 % annual rate in the UK [9] and far exceeds the 12.6 % half yearly rate (unpublished data) recorded among 749 Finnish participants in our OEV-123 ETEC vaccination trial [10].

### 5.2 Proportion of patients prescribed antibiotics at the primary visit

Of the 983 patients who visited the clinic for any reason, 398 (40.5 %) were prescribed antibacterial medication (Table 2), with 132 (33.2 %) of these receiving combination therapy (Table 3). This high prescribing rate is consistent with figures from other LMICs [11,12] and Pakistan [[13], [14], [15], [16]], exceeding the WHO-defined optimal range of 20–26.8 % [17] and surpassing those reported from high-income countries [18].

### 5.3 Most prescribed antibiotics

Metronidazole was the most common initial antibiotic, given to one third of patients, followed by first-generation cephalosporins (25 %), fluoroquinolones (23 %), and amoxicillin–clavulanate (21 %). To enable comparison with European community prescription data, we assessed antibiotic category proportions (ATC level 3) among our antibiotic prescriptions. The figures in our versus ECDC data [19] were for J01X (metronidazole) 24.2 % versus 5.6 %; for J01M (quinolones) 21.0 % versus 7.3 %; and for J01A (penicillins) 18.5 % versus 44.9 %. While ECDC uses DDD/1000/day and ours are prescription counts, the relative proportions remain broadly comparable.

These figures indicate substantial overuse of metronidazole and quinolones, consistent with other studies [16,20,21], and underprescription of penicillins. In Manga Mandi, over 90 % of penicillin prescriptions consisted of broad-spectrum amoxicillin–clavulanate, not phenoxymethylpenicillin or amoxicillin, which are most commonly prescribed, for example, in the UK [9].

In summary, prescribers relied heavily on metronidazole and broad-spectrum agents such as amoxicillin–clavulanate and fluoroquinolones, a pattern typical in Pakistan [16,20,21]. Stewardship interventions should promote substitution with narrow-spectrum alternatives when appropriate.

### 5.4 Appropriateness of antibiotic prescribing and drug choice

Given the data limitations, we applied a generous interpretation when evaluating both the decision to prescribe and the appropriateness of the chosen agent, assuming justification when uncertain.

Of the 398 antibiotic prescriptions, 218 (54.8 %) were prescribed to patients unlikely or highly unlikely to need antibiotics. While some in the ‘unlikely’ category may have benefited, this is offset by cases in the ‘possible’ category who did not (e.g. sore throat). We therefore estimate that at least 55 % were unnecessary. Regarding antibiotic choice (irrespective of need), fewer than 25 % aligned with first- or second-line recommendations, even under lenient criteria. Despite frequent use of broad-spectrum agents, nearly 20 % of prescriptions or combinations provided suboptimal coverage for the probable pathogens.

Overall, the data indicate widespread unnecessary antibiotic prescribing and, more critically, inappropriate drug selection, confirming the findings of earlier studies [16,20].

### 5.5 Antibiotics for common ailments

Among 76 patients presenting solely with sore throat, 58 (76.3 %) were prescribed antibiotics (Table 2), a higher proportion than previously reported in Pakistan [22] or elsewhere [23]. None received phenoxymethylpenicillin or amoxicillin, the first-line treatments for streptococcal pharyngitis [24], which, in the UK account for 80 % of sore throat prescriptions [23]. Instead, half received first-generation cephalosporins or macrolides, appropriate alternatives for penicillin-allergic patients [24], while the remainder obtained less appropriate agents, such as fluoroquinolones or metronidazole plus amoxicillin–clavulanate (Table 3).

Among the 80 patients with common cold or rhinitis symptoms, 55 (68.7 %) were prescribed antibiotics – similar to rates in other LMICs [12,25]. Most received first-generation cephalosporins, macrolides, and amoxicillin–clavulanate. Metronidazole and fluoroquinolones — drugs generally not recommended in upper respiratory infections [24] — were also common (Table 4). While most such cases are viral, some may represent bacterial sinusitis or pneumonia — conditions where amoxicillin is the preferred initial treatment [24]. Strikingly, amoxicillin was not prescribed at all, despite accounting for over half of respiratory antibiotic use in the UK [23].

Finally, among 55 patients seen solely by a dentist, 41 (74.5 %) received antibiotics, a striking contrast to <1 % in Wisconsin (USA); none received phenoxymethylpenicillin or amoxicillin, the standard first-line agents in dentistry [24,26].

### 5.6 Prescriptions at follow-up

Although the participants adhered meticulously to the scheduled follow-up timing, 233/493 (47.3 %) also made an unscheduled physician visit the same day – likely due to free consultations and the convenience of already being at the hospital. Nearly a third were prescribed antibiotics, raising concern over the necessity of both the visits and the prescriptions.

### 5.7 Factors contributing to overprescribing

Overprescribing and misprescribing in LMICs like Pakistan are driven by multiple factors: short consultations, patient expectations, knowledge gaps among physicians, limited diagnostic tools, medical culture, and pharmaceutical marketing [11,[27], [28], [29], [30]].

Our estimated average consultation time of 2.3 min is consistent with previous reports from Pakistan and other LMICs [[13], [14], [15],^28^]. Such brief visits limit history-taking, examination, and patient education, and are linked to increased antibiotic prescribing [11,27,28].

Laboratory and radiology services are often unavailable, forcing clinicians to make decisions based on limited data, often leading to pre-emptive antibiotic prescribing [30].

Patient expectations – real or perceived – are another driver [11,29,31]. In Punjab, over half of respondents believe that antibiotics are needed for common cold and any fever [32], these misconceptions cutting across education levels.

Pakistan's thriving pharmaceutical industry adds to the challenges: nearly 2000 antimicrobial brands compete in an aggressive market, with prescriber incentives common [32,33].

Finally, antibiotics are widely available over the counter, often sold by unqualified staff [32,33]. Nearly half of Punjabi university students had self-medicated with antibiotics within previous six months, most commonly metronidazole [34]. Rather than reduce the physician-focused reforms, this underscores how pharmacy practices often mirror prescriber behaviour [35].

### 5.8 Addressing over- and misprescription

Efforts to curb antibiotic over- and misprescribing often focus on staffing, diagnostics, and education [30]. Awareness campaigns and training or auditing interventions show promise but require time and resources [30,36]. Even simple algorithm-based tools – like the one reducing antibiotic use by 80 % in Tanzania [37] – can be difficult to implement due to short consultations. Interventions must work within existing infrastructure and budgets.

Given patient pressures and limited resources, shifting prescriptions towards more targeted agents may be a more feasible short-term goal than reducing overall use, [11,[28], [29], [30]]. For example, in our study, 76.3 % of sore throat patients were prescribed antibiotics, but none received phenoxymethylpenicillin, the first-line treatment. Instead, many received broad-spectrum antibiotics or metronidazole, which does not cover *Streptococcus pyogenes*. Defaulting to phenoxymethylpenicillin would reduce microbiota disruption and resistance without compromising outcomes or patient satisfaction.

Simplified prescription tools could help: while the WHO AWaRe manual spans 680 pages, its summary sheets [24] could be adapted into pocket cards or wall posters to offer quick, evidence-based support. Even if not comprehensive, such tools could offer a major step forward.

In summary, while systemic reforms remain the long-term goal [4], simple practical steps, like concise guidelines and focused training, could improve prescribing in LMICs and help in curbing AMR.

### 5.9 Strengths and weaknesses

This single-hospital snapshot is not meant to generalise but to ground global antibiotic consumption data in real-world, grassroot prescribing realities and behaviour.

Most global estimates rely on pharmaceutical sales data, particularly from the proprietary IQVIA-MIDAS database, which lacks methodological transparency and has patchy LMIC coverage [2,38,39]. Even if complete, sales figures alone cannot reflect clinical context.

Despite its single-clinic scope, this study provides such a reference point. By documenting real-world prescribing practices and thereby helping to interpret global figures.

A major limitation was the lack of detailed clinical data, mirroring systemic issues. Physicians had limited time, never documented diagnoses, and often prescribed without proper history or examination – constraints that mirrored our own. We relied on patient-reported symptoms, which likely matched the meagre information the physicians also had.

Patients may have obtained antibiotics from other doctors or without prescriptions. We believe this was uncommon since care and medications were free in Manga Mandi.

## 6 Conclusions

In this small outpatient clinic in Pakistan, we observed substantial overuse of antibiotics, including broad-spectrum antibiotic combinations, and metronidazole – even for upper respiratory tract infections.

We propose introducing simple guidelines to support rational prescribing. In Pakistan – and across South Asia, a known AMR hotspot [1,[3], [4], [5]], reducing the use of antibiotics, particularly broad-spectrum and anti-anaerobic agents [40], is imperative. However, as current efforts to limit antibiotic use in LMICs have met limited success [2,39], perhaps focusing on the *choice* of antibiotics rather than how *often* they are prescribed may prove more effective.

## CRediT authorship contribution statement

**Tamim Khawaja:** Conceptualization, Data curation, Formal analysis, Methodology, Writing – original draft, Writing – review & editing. **Mikael Kajova:** Writing – review & editing. **Anu Kantele:** Conceptualization, Methodology, Supervision, Writing – review & editing.

## Ethics approval and consent to participate

The study protocol was approved by the Ethics Committee of the School of Biological Sciences, University of the Punjab (SBS/822/15). Informed consent was obtained from all participants or their guardians.

## Funding

Finnish Government Funding for Health Science Research (VTR) (TYH2023309), the Sigrid Jusélius Foundation (1726), The University of Helsinki Doctoral School (TK), Inflammation Centre, HUS Helsinki University Hospital (TK) and the Finnish Multidisciplinary Centre of Excellence in Antimicrobial Resistance Research (FIMAR) funded by the Academy of Finland (346127).

## Declaration of competing interest

The authors declare the following financial interests/personal relationships which may be considered as potential competing interests:Anu Kantele reports financial support was provided by Finnish Government Funding for Health Science Research (VTR) (TYH2023309),. Anu Kantele reports financial support was provided by Sigrid Jusélius Foundation (1726). Anu Kantele reports financial support was provided by the Finnish Multidisciplinary Centre of Excellence in Antimicrobial Resistance Research (FIMAR) funded by the Academy of Finland (346127). Anu Kantele reports a relationship with Valneva Sweden AB that includes: funding grants. If there are other authors, they declare that they have no known competing financial interests or personal relationships that could have appeared to influence the work reported in this paper.
