# Supplementary material for: Improving antibiotic prescribing in LMICs: Insights from an outpatient clinic in Pakistan
Source: New Microbes New Infect. 2025 Dec 2;69:101680. doi: 10.1016/j.nmni.2025.101680 (PMC12754384; doi:10.1016/j.nmni.2025.101680)
Supplement: Multimedia component 1 [file mmc1.docx]

| **Supplementary Table 1**. Antibiotic agents^a^ prescribed in Manga Mandi during the study period | | | | | | |  |
| --- | --- | --- | --- | --- | --- | --- | --- |
| Antibiotic | Before Primary visit^b^, *n* | Primary visit, *n* (%) | Primary visit courses^d^, % | During the follow-up^c^, *n* | All prescriptions, *n* (%) | All courses^d^, % |  |
|  |  |  |  |  |  |  |  |
| FQ | 165 (22.7) | 93 (17.2) | 23.4 | 59 (23.4) | 317 (20.8) | 27.5 |  |
| MTZ | 163 (22.4) | 133 (24.5) | 33.4 | 69 (27.4) | 365 (24) | 31.6 |  |
| TET | 70 (9.6) | 63 (11.6) | 15.8 | 10 (4.0) | 143 (9.4) | 12.4 |  |
| ML | 59 (8.1) | 42 (7.7) | 10.6 | 17 (6.7) | 118 (7.8) | 10.2 |  |
| AMC | 131 (18.0) | 83 (15.3) | 20.9 | 47 (18.7) | 261 (17.1) | 22.6 |  |
| 1st CEP | 78 (10.7) | 100 (18.5) | 25.1 | 21 (8.3) | 199 (13.1) | 17.2 |  |
| 2nd/3rd CEP | 30 (4.1) | 12 (2.2) | 3 | 11 (4.4) | 53 (3.5) | 4.6 |  |
| 3rd CEP i.v. | 14 (1.9) | 6 (1.1) | 1.5 | 10 (4.0) | 30 (2) | 2.6 |  |
| AMX | 11 (1.5) | 1 (0.2) | 0.3 | 4 (1.6) | 16 (1.1) | 1.4 |  |
| SXT | 1 (0.1) | 0 (0) | 0 | 2 (0.8) | 3 (0.2) | 0.3 |  |
| RMP | 6 (0.8) | 5 (0.9) | 1.3 | 1 (0.4) | 12 (0.8) | 1 |  |
| GEN | 0 (0) | 1 (0.2) | 0.3 | 1 (0.4) | 2 (0.1) | 0.2 |  |
| PEN | 0 (0) | 2 (0.4) | 0.5 | 0 (0) | 2 (0.1) | 0.2 |  |
| SP | 0 (0) | 1 (0.2) | 0.3 | 0 (0) | 1 (0.1) | 0.1 |  |
| Total | 728 (100) | 542 (100) | NA | 252 (100) | 1522 (100) | NA |  |
| Abbreviations of antimicrobials: FQ = fluoroquinolones; MTZ = metronidazole; TET = tetracyclines; ML = macrolides; AMC = amoxicillin–clavulanate; 1st CEP = first-generation oral (p.o.) cephalosporins; 2nd/3rd CEP = second- or third-generation oral (p.o.) cephalosporins; 3rd CEP i.v. = third-generation parenteral cephalosporins; AMX = amoxicillin; SXT = trimethoprim–sulfamethoxazole; RMP = rifampin; GEN = gentamicin; PEN = parenteral benzylpenicillin; SP = sulfadoxine–pyrimethamine.  ^a^ Sulfadoxine/pyrimethamine and rifampin are included due to their notable activity against non-mycobacterial bacteria. Agents used primarily for antiparasitic or antimycobacterial indications were otherwise excluded. ^b^ Only the most recent antibiotic course prescribed in the six months preceding enrolment was recorded. ^c^ Refers to prescriptions issued between the primary visit and the 30-day control visit. Antibiotics prescribed on the day of the control visit are not included. ^d^ Columns labelled “courses” show the proportion of antibiotic courses containing the agent. | | | | | | |  |

| **Supplementary Table 2.** Antibiotic courses prescribed in Manga Mandi during the study period.**^a^** | | | | | |  |
| --- | --- | --- | --- | --- | --- | --- |
| Time of prescription | Patients with recorded antibiotic courses^b^ (*n*) | Antibiotic courses^b^ (*n*) | Antibiotic agents included in courses (*n*) | Monotherapy/ combination courses (*n*) | MTZ^b^ as monotherapy/ in combination (*n*) |  |
|  |  |  |  |  |  |  |
| Before primary visit^c^ | 571 | 571 | 728 | 423/148 | 31/132 |  |
| Primary visit | 398 | 398 | 542 | 266/132 | 14/119 |  |
| During the follow-up^d^ | 126 | 185 | 252 | 123/62 | 11/58 |  |
| Total | 1095 | 1154 | 1522 | 812/342 | 56/309 |  |
| ^a^A course of antibiotics is defined as the antibacterial agents prescribed at the same time^. b^MTZ ^=^ metronidazole. ^c^Only the most recent antibiotic prescription within 180 days prior to the primary visit was included for each patient.  ^d^Some patients received antibiotics on multiple occasions during the follow-up period. | | | | | |  |
